# Efficacy and safety of caffeic acid tablets in the treatment of thrombocytopenia: A systematic review and meta-analysis

**DOI:** 10.1097/MD.0000000000035353

**Published:** 2023-10-06

**Authors:** Hongxiu Yu, Ruixiang Chen, Zhengwen Zhou, Rongchun Liu, Jin Wen

**Affiliations:** a School of Pharmacy, Dali University, Dali, Yunnan, China; b Department of Pharmacy, The Third People’s Hospital of Yunnan Province, Kunming, Yunnan, China.

**Keywords:** caffeic acid tablets, efficacy, meta-analysis, safety, thrombocytopenia

## Abstract

**Background:**

Caffeic acid tablets (CFA) are a proprietary Chinese medicine in treating thrombocytopenia. The efficacy and safety of CFA compared with other platelet-raising drugs for the treatment of thrombocytopenia have been widely reported in the literature, but there is no systematic evaluation. Therefore, we designed this meta-analysis to further establish the efficacy and safety of CFA in treating thrombocytopenia.

**Methods:**

A computerized search was conducted in the Chinese biomedical database (CBM), Chinese National Knowledge Infrastructure (CNKI), Wanfang database, Chinese Scientific Journal Database (VIP), PubMed, and Web of Science databases using the keywords “caffeic acid tablets” and “thrombocytopenia.” All randomized controlled trials were selected for the timeframe of build to 02/2023 and then screened and analyzed using RevMan 5.4 and stata17.0 software.

**Results:**

A total of 35 publications with an overall 2533 patients were included in the study. The results of the meta-analysis showed that CFA were effective in the treatment of thrombocytopenia with a statistically significant difference [relative risk ratio (RR) = 1.24, 95% CI (1.17, 1.31), *P* < .00001] and in increasing platelet counts [standardized mean difference (SMD) = 1.50, 95% CI (1.09, 1.91), *P* < .00001], white blood cell count [SMD = 1.08, 95% CI (0.77, 1.39), *P* < .00001], and neutrophil count [SMD = 0.73, 95% CI (0.19, 1.28), *P* = .009], and CFA reduced myelosuppression [RR = 0.19, 95% CI (0.1, 0.37), *P* < .00001] and adverse effects [RR = 0.75, 95% CI (0.58, 0.96), *P* = .02].

**Conclusion:**

CFA can effectively improve the clinical outcome of patients with thrombocytopenia with a good safety profile and are worth promoting. However, due to the low quality and small sample size of the included literature, a larger sample size and more standardized, high-quality studies are needed to validate these results.

## 1. Introduction

Platelets are derived from megakaryocytes, and their production and maturation in the bone marrow is regulated by thrombopoietin.^[1]^ Platelets play an important role not only in thrombosis and wound repair, but also in inflammation, immunity and cancer biology.^[2]^ Thrombocytopenia refers to a platelet count (PLT) <100 × 10^9^/L. The main pathogenesis of thrombocytopenia is a decrease in platelet production, increased destruction and accumulation in the spleen, usually due to bacterial or viral infections, liver diseases, hematologic diseases, malignant tumors, pregnancy, autoimmune diseases, thrombotic microangiopathy, etc.^[3]^ Thrombocytopenia is a common problem that affects 40% to 50% of medical and surgical intensive care units.^[4]^ For thrombocytopenia caused by immune thrombocytopenic purpura (ITP), the main first-line treatment is glucocorticosteroids, immunoglobulins, etc.^[5–7]^ Second-line treatment strategies include thrombopoietin receptor agonists; however, these agents often fail to achieve durable remission and require additional treatment options.^[8]^ For chemoradiotherapy-induced thrombocytopenia, treatment includes platelet transfusions and administration of platelet growth factors, but the side effects are numerous and expensive.^[9]^

The main ingredient of caffeic acid tablets (CFA) is caffeic acid (CA). CA is a hydroxycinnamic acid that belongs to the phenolic acid family of polyphenols. And also known as “3,4-hydroxycinnamic acid” or “3,4-dihydroxy phenyl acrylic acid.”^[10]^ Its molecular formula: C_9_H_8_O_4_, relative molecular weight: 180.16 Da.^[11]^ It is the main hydroxycinnamic acid present in the human diet, with the highest content being found in blueberries, kiwis, plums, cherries, and apples, although also present in cereals, carrots, salad, eggplants, cabbage, artichoke, and coffee.^[12–14]^ In vitro and in vivo experiments have been performed, proving innumerable physiological effects of CA and its derivatives, such as antibacterial activity,^[15,16]^ antiviral activity,^[17–20]^ antioxidant activity,^[16–20]^ anti-inflammatory activity,^[16–20]^ anti-atherosclerotic activity,^[15,16]^ immunostimulatory activity,^[15,21]^ antidiabetic activity,^[17,20]^ cardioprotective activity,^[17,22]^ antiproliferative activity,^[15,22,23]^ hepatoprotective activity,^[24,25]^ anticancer activity,^[16–20]^ and anti-hepatocellular carcinoma activity.^[26–28]^ Besides these important activities, CA and its derivatives have shown a very high potential for treating and preventing cardiovascular and cancer diseases in preclinical studies.^[10,29]^ In addition, CA also has the effects of hemostasis, raising white blood cells and platelets, and is clinically used in the treatment of leukopenia and thrombocytopenia caused by various reasons.^[30]^

The efficacy and safety of CFA compared with other platelet-raising drugs in the treatment of thrombocytopenia have been widely reported in the literature, but there is no systematic evaluation, so this study used meta-analysis to compare the efficacy and safety of CFA with other platelet-raising drugs in the treatment of thrombocytopenia, with the aim of providing a reliable basis for clinical use and evidence-based guidelines.

## 2. Methods

### 2.1. Ethical approval and consent to participate

The PRISMA guidelines^[31]^ were used for designing and reporting this study, and this study was in accordance with the ethical guidelines of the Declaration of Helsinki (as revised in 2013).^[32]^This study is a systematic evaluation type article and does not require ethical approval.

### 2.2. Literature search strategy

Four Chinese databases (CNKI, Sinomed, VIP, and Wanfang) and 4 English databases (Web of Science, PubMed, Embase, and Cochrane Library) were searched for the terms: “Caffeic acid”, “Caffeic acid tablet”, “Thrombocytopenia”, “Thrombocytopenias”, “Thrombopenia”, “Thrombopenias”, and “randomized controlled trial,” in both Chinese and English. The search time frame was established for each database until September 2022.

### 2.3. Inclusion criteria

Research type: Only randomized controlled trials were included and the languages were limited to Chinese and English. Research object: The included study cases were all patients with a diagnosis of thrombocytopenia. Refer to Consensus on the clinical diagnosis, treatment, and prevention of chemotherapy-induced thrombocytopenia in China (2019 version),^[9]^ Thrombocytopenia is classified as degree I–IV, degree I: (50–100) × 10^9^/L; degree II: (30–50) × 10^9^/L; degree III: (20–30) × 10^9^/L; degree IV: less than 10 × 10^9^/L. Patients with a white blood cell count below 100 × 10^9^/L can be diagnosed with thrombocytopenia, and the patient age and gender are not limited. Intervention measures: Patients in the test group were treated with CFA (200–300 mg/time, 3 times/d. Dezhou Deyao Pharmaceutical Co., Ltd., approved by Chinese medicine H37020537, production batch number: 2102140503.) or CFA combined with conventional platelet-raising drugs, while the control group was treated with conventional drugs (hormonal drugs, recombinant human granulocyte-stimulating factor and other platelet-raising drugs), and the dose and duration of administration were not limited. Outcome indicators: The primary indicators were PLT changes and clinical efficacy (complete remission: PLT ≥ 100 × 10^9^/L; partial remission: 50 × 10^9^/L < PLT < 100 × 10^9^/L; ineffective: PLT ≤ 50 × 10^9^/L, clinical efficacy = complete remission + partial remission). Secondary indicators were incidence of adverse reactions, bone marrow suppression, change in white blood cell count, change in neutrophil count, and change in hemoglobin.

### 2.4. Exclusion criteria

Have any serious medical condition such as heart, brain, and kidney injury; no studies of any of the outcome indicators included in this study or literature not available in full; no control group or self-control; cohort studies, animal studies, clinical experience, etc.; and conference articles.

### 2.5. Literature screening and data extraction

The literature was screened and cross-checked by 2 investigators independently and in case of disagreement, both parties negotiated and requested a ruling from a third investigator. Information extracted from the literature included the first author, year of publication, type of disease, number of patients, mean age, interventions, outcome indicators, and duration of treatment.

### 2.6. Literature quality assessment

The quality of the included literature was evaluated using the risk of bias assessment tool recommended in the Cochrane Systematic Evaluator Handbook 5.1.0. This specifically included random sequence generation, allocation concealment, blinding, completeness of outcome data, and other sources of bias. Each item was categorized into content rated as high risk, unclear and low risk.^[33]^

### 2.7. Statistical methods

Meta-analysis was performed using RevMan 5.3 and Stata 17.0 software. Count data were analyzed by relative risk ratio (RR), and continuous data were analyzed by mean difference (MD) or standardized mean difference (SMD). Also, their combined effect sizes and their 95% confidence intervals (CI) were calculated. Heterogeneity was analyzed using the Q test and I2 test.^[34]^ If there was no statistical heterogeneity between studies (I2 < 50%, *P* > .10), a fixed-effects model was used for analysis, otherwise, a random-effects model was used. The same outcome indicators were analyzed in subgroups according to disease type, treatment regimen, and the duration of treatment. Sensitivity analysis was performed using Stata 17.0 software. The publication bias analysis was performed using inverted funnel plots. *P* < .05 was considered statistically significant.

## 3. Results

### 3.1. Literature screening results

**Figure 1.**
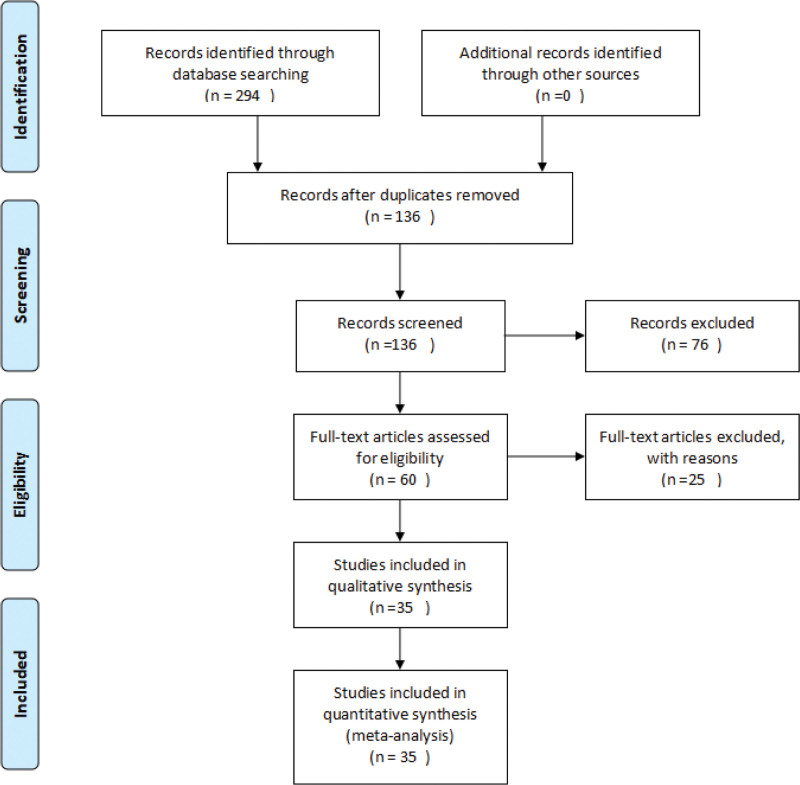
Flowchart of literature screening.

A total of 294 pieces of relevant literature were obtained from the initial review and after reading the title, abstract and full text of 35 studies with a total of 2533 patients including 1278 in the trial group and 1255 in the control group according to the inclusion and exclusion criteria. The literature screening process has been done as shown in Figure 1.

### 3.2. Basic characteristics of the included literature

The basic information of the literature included were shown in Table 1.^[20]^ Most of the patients were cancer patients with the youngest participant being 7 years old and the oldest being 62 years old. The shortest duration of treatment in the study was 2 weeks and the longest was 12 weeks.

**Table 1:** Basic information of included studies.

Author/yr	Disease type	Age (yr)		Platelet count ($\bar{\text{x}}\pm \text{s}$, x10^9^/L)		Number of cases		Intervention		Treatment (wk)	Outcome indicators
		T	C	T	C	T	C	T	C		
Beiyuexian, 2015^[35]^	ITP	35.7 ± 12.4	33.42 ± 10.2	156.9 ± 52.4	141.5 ± 61.7	55	55	CFA	Hormone therapy	4	(a)(b)(c)
Chenming, 2012^[36]^	ITP	35.2	33.3	81.5 ± 16.48	69.5 ± 5.42	41	23	CFA	Hormone therapy	5	(a)
Donghanzhi, 2020^[37]^	Squamous lung cancer	54	54	189.22 ± 55.86	156.38 ± 59.88	25	25	CFA	Conventional chemotherapy	2	(a)(d)
Duzunming A., 2018^[38]^	Lymphoma	56	55	173.94 ± 51.7	123.85 ± 46.67	41	41	CFA	G-CSF	3	(a)(c)(e)(f)
Duzunming B., 2018^[39]^	Lung cancer	62	60	217.26 ± 19.74	111.59 ± 20.41	60	60	CFA	G-CSF	4	(a)(b)(c)(e)(f)
Hedi, 2020^[40]^	ITP	46.28 ± 5.19	48.79 ± 6.14	121.56 ± 21.95	78.22 ± 16.44	52	52	CFA	Hormone therapy	4	(a)(b)(c)
Huangzhenhua, 2013^[41]^	Uremia	50.8	49.6	193.61 ± 11.03	99.68 ± 12.09	12	12	CFA	Conventional Hemostatic Drugs	4	(a)
Jiaxiaohui, 2013^[42]^	Systemic lupus erythematosus	35.2 ± 18.4	36.8 ± 16.7	97 ± 38.5	72 ± 36.7	30	30	CFA	Leucogen Tablets	4	(a)(d)(e)
Kangjide, 2013^[43]^	ITP	30.9 ± 10.8	31.5 ± 7.5	144.6 ± 45.3	132.5 ± 50.8	45	45	CFA	Hormone therapy	4	(a)(b)(c)
Linwei, 2022^[44]^	Leukemia	16.82 ± 3.32	17.73 ± 3.07	57.86 ± 4.72	38.25 ± 3.94	40	40	CFA	rhIL-11	2	(a)(c)(d)(e)
Linwenyuan, 2019^[45]^	ITP	36	36	147.3 ± 69.8	138.3 ± 58.2	34	41	CFA	Hormone therapy	8	(a)(b)
Liudan, 2015 ^[46]^	ITP	30.8 ± 5.1	31.4 ± 5.2	NR	NR	26	26	CFA	Hormone therapy	6	(b)(c)
Liufeng, 2011^[47]^	Aplastic anemia	33	33	76 ± 15	50 ± 14	49	43	CFA	Hormone therapy	12	(a)(b)(e)
Liuke, 2012 ^[48]^	ITP	30	30	145 ± 60.1	140 ± 50.1	49	49	CFA	Hormone therapy	8	(a)
Liuxueru, 2016^[49]^	Esophageal cancer	58	56	NR	NR	24	24	CFA	Leucogen Tablet Vitamin B tablet	4	(b)(c)(e)
Lixiansong, 2013^[50]^	ITP	35	35	NR	NR	14	14	CFA	Anti-HP conventional treatment drugs	4	(b)
Lixiaohong, 2013^[51]^	ITP	65	65	86.00 ± 44.74	64.26 ± 29.22	31	31	CFA	Herbs for clearing heat and cooling the blood	12	(a)(b)
Luoyihui, 2013^[52]^	Cirrhosis of the liver	56	56	NR	NR	50	50	CFA	Leucogen Tablet	4	(b)
Majun, 2017 ^[53]^	ITP	32.9	32.9	79.90 ± 43.95	57.87 ± 24.25	60	60	CFA	Coffee acid tablet mockups	4	(a)(b)(c)
Mengdeqian, 2018^[54]^	Systemic lupus erythematosus	39.4 ± 17.6	37.3 ± 14.1	128.70 ± 52.55	102.50 ± 35.70	31	32	CFA	Hormone therapy	4	(a)(c)(e)
Mengdeqian, 2021^[55]^	Dryness syndrome	44.6 ± 11.7	42.4 ± 10.5	156.03 ± 87.92	123.72 ± 64.38	36	36	CFA	Hormone therapy	4	(a)(c)(e)
Miudong, 2013^[56]^	ITP	41.32 ± 7.38	41.32 ± 7.38	NR	NR	15	15	CFA	Conventional chemotherapy	NR	(b)
Panbo, 2013 ^[57]^	Chemotherapy prevention	44.6 ± 11.7	42.4 ± 10.5	178.8 ± 40.3	89.4 ± 30.5	41	41	CFA	Conventional chemotherapy	3	(a)
Pangying, 2012^[58]^	lymphadenoma	55.6	53	223 ± 121	143.5 ± 94.5	30	30	CFA	Batilol tablet	2	(a)(c)(e)
Pengaihua, 2015^[59]^	Breast Cancer	48 ± 8.47	49 ± 9.37	327 ± 67.55	274 ± 48.02	30	30	CFA	G-CSF	3	(a)(c)(d)(e)(f)
Tangtianbi, 2017^[60]^	Tuberculosis	43.4 ± 12.3	41.4 ± 11.2	118.77 ± 39.72	93.23 ± 19.29	29	28	CFA	Leucogen Tablet	3	(a)(b)(c)
Wanghaicun, 2016^[61]^	Breast Cancer	44.23 ± 6.26	45.49 ± 6.51	319.00 ± 72.32	286.00 ± 62.52	40	40	CFA	G-CSF	2	(a)(b)(d)(e)
Wangjun, 2013^[62]^	ITP	NR	NR	113 ± 26.61	79.7 ± 27.36	20	20	CFA	Hormone therapy	4	(a)
Xinxiaohai, 2015^[63]^	Chemotherapy prevention	53.2 ± 2.1	53.2 ± 2.1	178.9 ± 40.2	89.2 ± 30.4	40	40	CFA	Conventional chemotherapy	3	(a)
Xudan, 2019 ^[64]^	ITP	42.7 ± 3.1	44.8 ± 4.4	NR	NR	40	40	CFA	Leucogen Tablet	12	(b)
Yangxi, 2019 ^[65]^	Children ITP	7.1 ± 0.6	7.2 ± 0.8	91.52 ± 21.31	80.88 ± 20.65	49	49	CFA	Hormone therapy	4	(a)(b)
Zhangxiaomeng, 2022^[66]^	Hematologic Tumors in children	7.5 ± 2.31	7.5 ± 2.61	201.25 ± 45.52	152.25 ± 41.52	49	49	CFA	G-CSF	2	(a)(c)(d)(e)(f)
Zhangxuejuan, 2013^[67]^	ITP	37.3 ± 10.92	36.7 ± 9.17	86 ± 12	36 ± 11	33	27	CFA	Hormone therapy	4	(a)(b)
Zhaobianfeng, 2017^[68]^	Lung cancer	55.1	54.3	227.9 ± 100.5	193.5 ± 84.2	32	32	CFA	Batilol tablet	2	(a)(c)(e)
Zhoucaixia, 2022^[69]^	Aplastic anemia	37.58 ± 6.89	36.48 ± 6.43	81.39 ± 0.54	67.84 ± 2.58	25	25	CFA	Hormone therapy	12	(a)(b)

T is the test group and C is the control group. Outcome indicators: (a) platelet count; (b) clinical efficacy; (c) incidence of adverse effects; (d) neutrophil count; (e) leucocyte count; and (f) myelosuppression.

CFA = control group on the basis of caffeic acid tablets, G-CSF = recombinant human granulocyte colony-stimulating factor, ITP = immune thrombocytopenic purpura, NR = not reported, rhIL-11 = platelet-producing drugs.

### 3.3. Risk of bias assessment for inclusion in the literature

**Figure 2.**
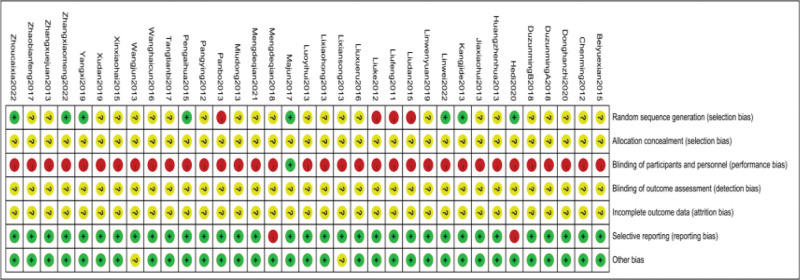
Risk of bias map.

**Figure 3.**
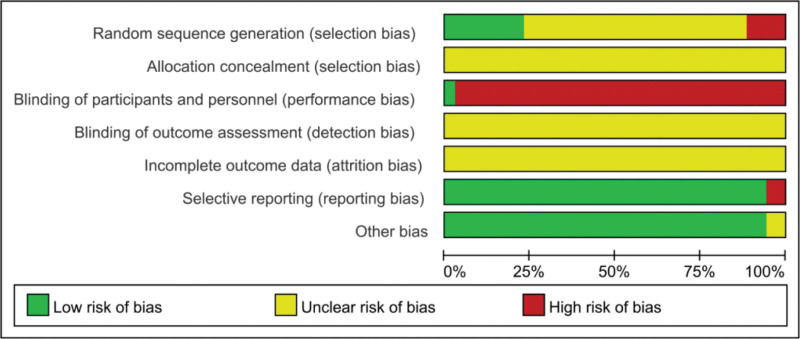
Risk of bias bar chart.

All included trials were found to have a high risk of bias due to inadequate or insufficient reporting of information on study design and methods. All studies were randomized controlled trials, However, only 12 studies^[40,43,44,46–48,53,57,59,65,66,69]^elucidated the method of random sequence generation, Five of them^[40,43,44,59,65]^used the random number table method, one^[66]^used the computerized randomization method, one^[69]^used the touch ball method, and one^[53]^followed the stratified block group to generate the random assignment and were therefore assessed as having a low risk of bias. Four items^[46–48,57]^described the use of visit order to generate random assignment and were therefore assessed as having a high risk of bias. Since CFA were only used in the trial group, it seems unlikely that any of the trials blinded participants and staff, except for Majun 2017^[53]^which used double blinding. Information on allocation concealment and blinding of outcome assessment was not reported in any of the trials and was therefore judged as unclear. Withdrawal information was not reported in any of the trials and was therefore assessed as unclear. As none of the trials provided information on trial registration, we assessed reporting bias by judging the consistency between results in the methods section of the publication, and 2 of the trials^[40,54]^were assessed to be at high risk of selective reporting bias because it had apparent problems with primary outcome reporting. The remaining trials were assessed as having a low risk of selective reporting bias due to studies reporting all outcomes mentioned in the methods section. Other bias was assessed by comparability of baseline data between the 2 groups, with 2^[51,62]^trials reporting baseline data, including age and sex, with no statistical description of comparability, and therefore they were assessed as having an unclear risk of bias. See Figures 2 and 3 for details.

### 3.4. Results of meta-analysis

#### 3.4.1. Analysis of platelet count change and publication bias analysis.

**Figure 4.**
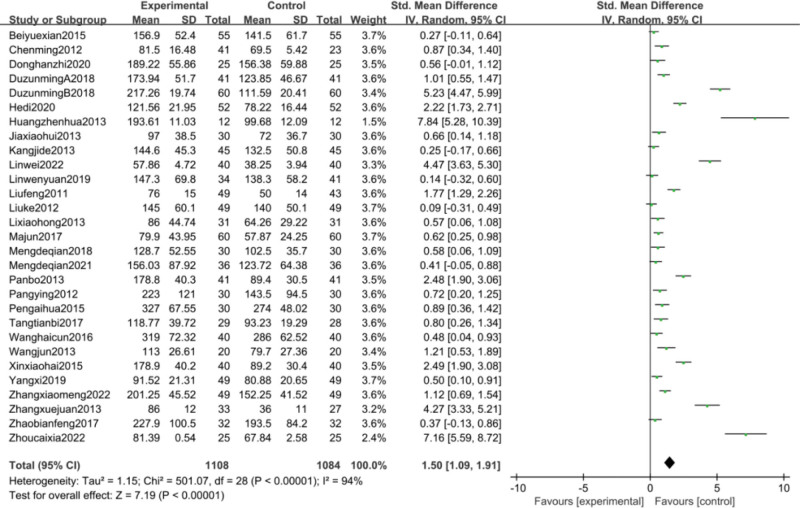
Meta-analysis of platelet count.

**Figure 5.**
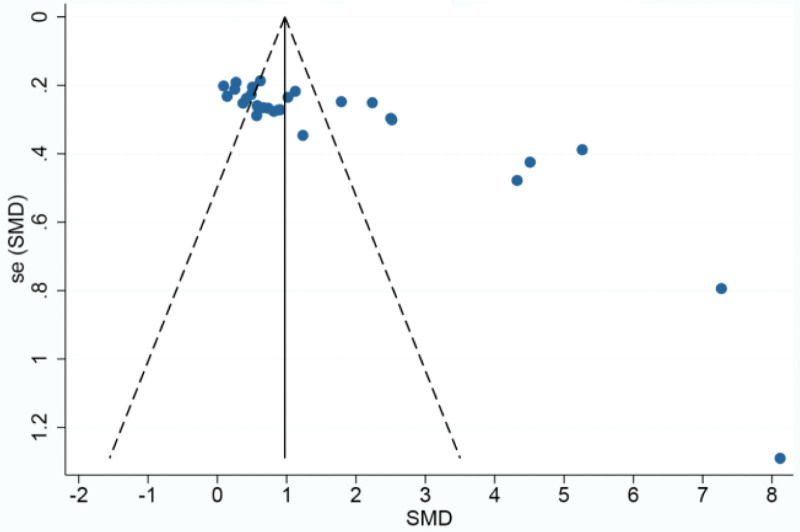
Funnel plot of platelet count.

Platelet counts were counted in 29^[35–45,47,48,51,53–55,57–63,65–69]^papers and were analyzed using a random-effects model because of the high heterogeneity between groups (*P* < .00001, I^2^ = 94%). Meta-analysis showed a statistically significant improvement in platelet counts in the CFA group compared with the control group [SMD = 1.50, 95% CI (1.09, 1.91, *P* < .00001)]. SMD = 1.50, 95% CI (1.09, 1.91), *P* < .00001] as detailed in Figure 4. Given the large heterogeneity, subgroup analysis was performed according to disease type, treatment regimen, and duration of treatment. The results of the subgroup analysis are detailed in Table 2. based on these results, it can be demonstrated that the source of heterogeneity may be related to the type of disease, but may not be related to the treatment regimen or the duration of treatment of the disease. Based on the changes in platelet counts, funnel plots were drawn using stata17.0 software with the standard error SE (SMD) of the effect size as the vertical coordinate and the SMD of the effect size for each study as the horizontal coordinate in Figure 5, which showed that the funnel plots were less symmetrical on both sides and there was a significant publication bias.

**Table 2:** Platelet count subgroup analysis.

Indicators	Number of included studies	SMD (95% CI)	Heterogeneity test		Effect model	*P*	Heterogeneity test	
			P	I^2^(%)			*P*	I^2^(%)
Oncological diseases	11^[37–39,44,57–59,61,63,66,68]^	1.77 (0.99, 2.55)	*P* < .00001	96	Random effects model	*P* < .00001	*P* = .04	68.8
ITP	11^[35,36,40,43,45,48,51,53,62,65,67]^	0.93 (0.44, 1.41)	*P* < .00001	92	Random effects model	*P =* .0002		
Other diseases	7^[41,42,47,54,55,60,69]^	2.17 (1.15, 3.18)	*P* < .00001	94	Random effects model	*P* < .0001		
Hormone therapy group	13^[35,36,40,43,45,47,48,54,55,62,65,67,69]^	1.33 (0.75, 1.90)	*P* < .00001	94	Random effects model	*P* < .00001	*P* = .43	0
Non-hormonal therapy group	16^[37–39,41,42,44,51,53,57–61,63,66,68]^	1.65 (1.07, 2.24)	*P* < .00001	95	Random effects model	*P* < .00001		
≤2 wk	6^[37,44,58,61,66,68]^	1.24 (0.38, 2.09)	*P* < .00001	94	Random effects model	*P* = .005	*P* = .48	0
>2 wk	23^[35,36,38–43,45,47,48,51,53–55,57,59,60,62,63,65,67,69]^	1.59 (1.11, 2.06)	*P* < .00001	95	Random effects model	*P* < .00001		

ITP = immune thrombocytopenic purpura, SMD = standardized mean difference.

#### 3.4.2. Clinical efficacy analysis and publication bias analysis.

**Figure 6.**
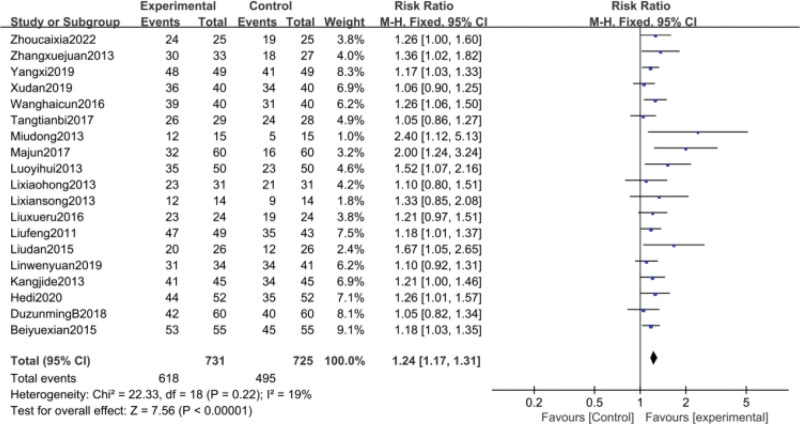
Meta-analysis of clinical efficacy.

**Figure 7.**
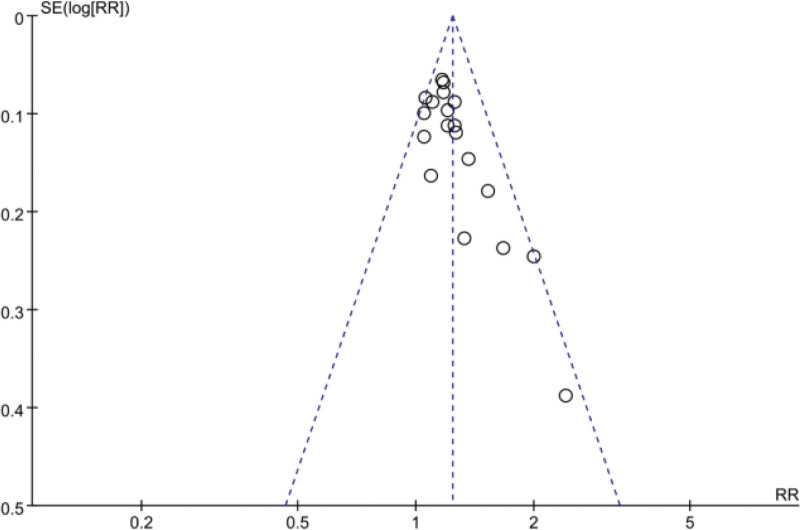
funnel plot of clinical efficacy.

Clinical efficacy was counted in 19^[35,39,40,43,45–47,49–53,56,60,61,64,65,67,69]^papers and was analyzed using a fixed-effects model because of high heterogeneity between groups (*P* = .22, I^2^ = 19%). Meta-analysis showed that the clinical efficacy of the trial group for thrombocytopenia was better than that of the control group, and the difference was statistically significant [RR = 1.24, 95% CI (1.17, 1.31), *P* < .00001] Figure 6. its funnel plot is shown in Figure 7, the 2 sides of the funnel plot are not quite symmetrical, suggesting a possible publication bias.

#### 3.4.3. White blood cell count analysis.

**Figure 8.**
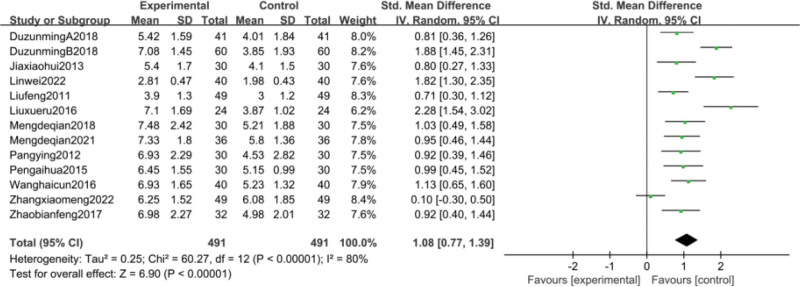
Meta-analysis of WBC.

**Figure 9.**
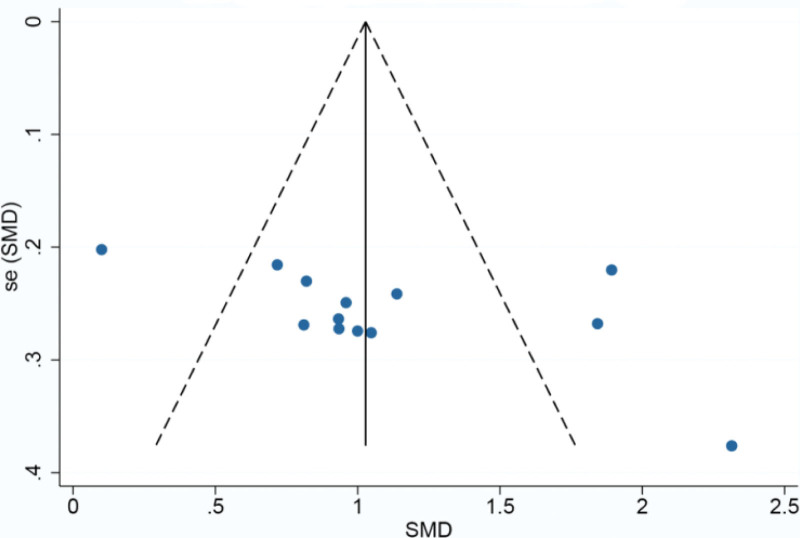
funnel plot of WBC.

Thirteen studies^[38,39,42,44,47,49,54,55,58,59,61,66,68]^reported changes in leukocyte counts before and after treatment. Meta-analysis results showed high heterogeneity (*P* < .00001, I^2^ = 80%) between study groups, so random-effects model analysis was used. Meta-analysis showed that compared with the control group CFA group significantly improved PLT with a statistically significant difference [SMD = 1.08, 95% CI (0.77, 1.39), *P* < .00001] as detailed in Figure 8. considering the high heterogeneity, subgroup analysis was performed according to disease type, treatment regimen and duration of treatment. The results of the subgroup analysis are detailed in Table 3. Based on these results, it can be demonstrated that the source of heterogeneity may be related to the type of disease, but may not be related to the treatment regimen or the duration of treatment of the disease. Based on the changes in leukocyte counts, funnel plots were drawn using stata17.0 software with the standard error SE (SMD) of effect sizes as the vertical coordinate and the SMD of effect sizes for each study as the horizontal coordinate in Figure 9, which showed that the funnel plots were less symmetrical on both sides and that there was publication bias.

**Table 3:** Leukocyte count subgroup analysis.

Indicators	Number of included studies	SMD (95% CI)	Heterogeneity test		Effect model	*P*	Heterogeneity test	
			*P*	I^2^(%)			*P*	I^2^(%)
Oncological diseases	9^[44,48,49,58,61,66,68]^	1.18 (0.74, 1.62)	*P* < .00001	86	Random effects model	*P* < .00001	*P* = .20	38.4
Other diseases	4^[42,47,54,55]^	0.86 (0.61, 1.10)	*P* = .79	0	Random effects model	*P* < .00001		
Hormone therapy group	5^[38,39,59,61,66]^	0.98 (0.37, 1.59)	*P* < .00001	89	Random effects model	*P* = .002	*P* = .64	0
Non-hormonal therapy group	8^[42,44,47,49,54,55,58,68]^	1.15 (0.81, 1.48)	*P* = .002	69	Random effects model	*P* < .00001		
≤2 wk	5^[44,58,61,66,68]^	0.97 (0.39, 1.54)	*P* < .0001	86	Random effects model	*P* = .001	*P* = .59	0
>2 wk	8^[38,39,42,47,49,54,55,59]^	1.15 (0.79, 1.51)	*P* = .0002	76	Random effects model	*P* < .00001		

SMD = standardized mean difference.

#### 3.4.4. Analysis of neutrophil count.

**Figure 10.**
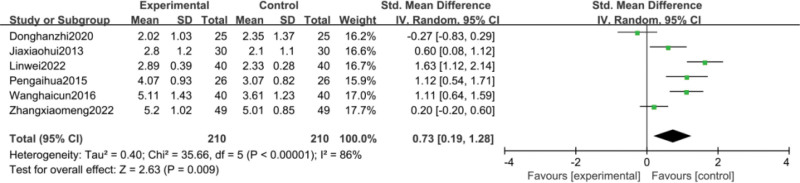
Meta analysis of neutrophil counts.

Six studies^[37,42,44,59,61,66]^reported changes in neutrophil counts before and after treatment. Meta-analysis results showed a statistically significant improvement in neutrophil counts in the CFA group compared to the control group [SMD = 0.73, 95% CI (0.19, 1.28), *P* = .009], with heterogeneity (*P* < .00001, I^2^ = 86%), so a random-effects model analysis was used as detailed in Figure 10.

#### 3.4.5. Myelosuppression rate at degrees III and IV.

**Figure 11.**
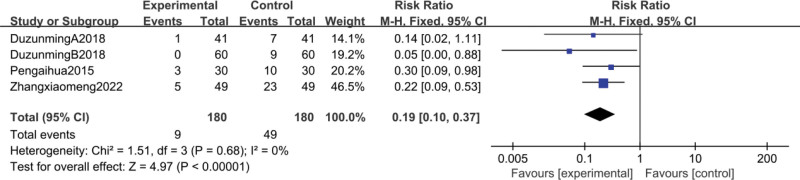
Meta-analysis of grade III and IV myelosuppression.

Four studies^[38,39,59,66]^reported III and IV degrees of myelosuppression, and the meta-analysis showed that the CFA group may reduce the occurrence of myelosuppression compared to the control group [RR = 0.19, 95% CI (0.1, 0.37), *P* < .00001], heterogeneity (*P* = .68, I^2^ = 0%), so a fixed-effect model analysis, as in Figure 11.

#### 3.4.6. The incidence of adverse reactions.

Adverse reactions were reported in 12 studies^[38–40,43,44,49,54,55,59,60,66,68]^ and the details of the included adverse reactions are shown in Table 4, while the others did not report the specifics of the adverse reactions. These results suggest that CFA have no or mostly mild adverse reactions in patients.

**Table 4:** The occurrence of adverse reactions.

Author/yr	Adverse reaction	
	T	C
Duzunming A., 2018^[38]^	4 cases of nausea and epigastric discomfort, 1 case of diarrhea, 2 cases of mildly elevated alanine aminotransferase, 6 cases of bleeding, and 10 cases of infection	15 cases of bleeding, 23 cases of infection
Duzunming B., 2018^[39]^	2 cases of nausea and epigastric discomfort, 1 case of mild diarrhea, and 1 case of mildly elevated alanine aminotransferase	0
Hedi, 2020^[40]^	6 cases of nausea, 1 case of renal dysfunction, 2 cases of peptic ulcer	5 cases of nausea, 2 cases of renal dysfunction, 1 case of peptic ulcer
Kangjide, 2013^[43]^	5	4
Linwei, 2022^[44]^	1 case of dizziness, 2 cases of headache, 1 case of fever, 1 case of malaise	1 case of dizziness, 1 case of fever, 1 case of malaise
Liuxueru, 2016^[49]^	1 case of mildly elevated alanine aminotransferase and 1 case of nausea	4
Mengdeqian, 2018^[54]^	1 case of epigastric discomfort, nausea	0
Mengdeqian, 2021^[55]^	3 cases of nausea, nausea	2 cases of loss of appetite
Pengaihua, 2015^[59]^	1 case of abnormal liver function, 2 cases of abnormal electrocardiogram	2 cases of abnormal liver function, 1 case of abnormal renal function, 3 cases of abnormal electrocardiogram, 1 case of infection
Tangtianbi, 2017^[60]^	3 cases of abnormal liver function	3 cases of abnormal liver function
Zhangxiaomeng, 2022^[66]^	1 case of local reaction, 2 cases of dizziness	1 musculoskeletal, 2 local reactions, 2 fever, 2 dizziness, 1 other
Zhaobianfeng, 2017^[68]^	2 cases of skin bleeding, 2 cases of urinary tract infection, 3 cases of respiratory tract infection	2 cases of rhinorrhea, 3 cases of skin bleeding, 2 cases of urinary tract infection, 7 cases of respiratory tract infection

**Figure 12.**
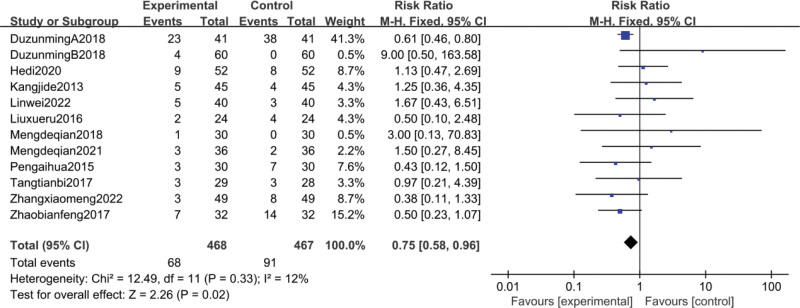
Meta-analysis of adverse reactions rate.

12 studies^[38–40,43,44,49,54,55,59,60,66,68]^ reported the incidence of adverse reactions in patients after treatment. the meta-analysis showed heterogeneity (*P* = .33, I^2^ = 12%), so a fixed effects model was used to analyze the results. The results showed that the CFA group had a better safety profile compared to the treatment in the control group [RR = 0.75, 95% CI (0.58, 0.96), *P* = .02] and the difference was statistically significant, as shown in Figure 12.

#### 3.4.7. Sensitivity analysis.

Sensitivity analysis of the included studies has shown no significant differences between the random-effects model and fixed-effects model. The exclusion of any of the studies had little effect on the results indicating low sensitivity and strong confidence in the results.

## 4. Discussion

Thrombocytopenia, usually defined as a PLT of < 100 × 10^9^/L in peripheral blood, is a common complication in oncology patients.^[70]^ However, ITP can also present with symptoms of thrombocytopenia. ITP is a hemorrhagic disease characterized by an increase in platelet destruction due to the production of anti-auto-platelet antibodies in the patient body, resulting in a persistent reduction of platelets in the peripheral blood and a normal or increased number of bone marrow megakaryocytes with maturation disorders, and it is the most common type of thrombocytopenic disease in the clinic.^[71]^Thrombocytopenia may increase a patient risk of bleeding, thereby endangering the patient life and health, as well as affecting treatment outcomes and increasing medical costs.^[72]^ Thrombocytopenia is mostly treated with glucocorticoids, splenectomy and immunomodulators vincristine, cyclophosphamide and rituximab in western medicine, which have greater adverse effects. Adverse effects of glucocorticoids include osteoporosis and even aseptic femoral head necrosis, water and sodium retention, centripetal obesity, acne and elevated blood pressure and blood glucose, which have a high incidence and seriously affect the quality of life of patients.^[63]^

CFA are rapidly absorbed after oral administration and have a high distribution in the blood and kidneys. The mechanism of action of CFA is complex and varied^[73,74]^, Ferulic acid and isoferulic acid are the 2 main bioactive metabolites of caffeic acid. The t_1/2_ of caffeic acid was 1.25 ± 0.26 hours. The reason for the difference in t_1/2_ of caffeic acid in different pathways may be the rapid transfer from blood to tissue. Its absolute bioavailability was 3.4%, suggesting that caffeic acid may have a rapid biotransformation in vivo, with poor permeability through the intestinal epithelial membrane.^[75]^ Caffeic acid also stimulates the differentiation of hematopoietic stem cells, the proliferation of endothelial cells and the expression of surface adhesion molecules, thus improving the hematopoietic function of the body. It also increases the synthesis of deoxyribonucleic acid in leukocytes, stimulates the synthesis of megakaryocyte proteins and antioxidants, which contribute to anti-apoptosis, regulates the function of T cells and reduces the destruction of peripheral blood cells, thus increasing the level of platelets and leukocytes.^[65]^ Caffeic acid also improves microvascular and platelet function and coagulation factors by reducing the time to coagulation and hemostasis.^[76]^ In patients with thrombocytopenia and leukopenia, caffeic acid has been found to have a rapid onset of action and precise efficacy.^[77,78]^

In this study, 35 papers were included through a search of relevant domestic and international literature, and the results of a clinical randomized controlled study comparing the CFA group with the non-CFA group by meta-analysis showed that the difference between the CFA group and the control group was statistically significant. From a clinical perspective, CFA did increase platelet counts and improve patient health. In addition, there were few serious adverse events and treatment-related safety issues, and our subgroup analysis of 2 outcome indicators, PLT and white blood cell count, showed that the classification of the disease may be of heterogeneous origin. The results of this study showed that the application of CFA for the treatment of thrombocytopenia does have good clinical efficacy, and that CFA are safe and effective, have mild adverse effects, are inexpensive and easy to use, and are clinically worth promoting. From the results of the funnel plot, it can be seen that there may be publication bias in the meta-analysis, and from the sensitivity analysis showed that the sensitivity analysis of each index was consistent with the original results, with good stability and high confidence of the conclusion.

Our study still has some limitations. Firstly, the included articles were all Chinese studies with geographical limitations. Secondly, the sample size of each study was mostly from 1 hospital, which is a small sample size that may affect the reliability and accuracy of the findings. Furthermore, only 12 of the 35 papers reported a specific randomization method, and 4 of them were high-risk biased. Since some of the original studies were published too early, many did not report the use of allocation concealment and blinding and therefore their quality was not high. However, the aim of this study was to investigate the effect of CFA on thrombocytopenia. We chose objective results from clinical trials, such as PLT, white blood cell count, and neutrophil count, which were minimally affected by allocation concealment and blinding. Second, the heterogeneity between studies cannot be ignored because of the different interventions, drug doses, and treatment durations used in patients with different platelet levels in each trial. In our study, we performed a subgroup analysis to reduce heterogeneity to some extent. Finally, for adverse events, there were no clear conclusions about adverse effects of CFA due to the limited follow-up time of the included studies and the non-standardized reporting of some studies.

## 5. Conclusion

The results of this study showed that CFA are effective, safe and economical in the treatment of thrombocytopenia and are worthy of clinical application. However, due to the limitations of the quality of the included literature, sample size and study design, the above findings need to be validated by further large sample and high-quality clinical studies in the future.

## Author contributions

**Methodology:** Zhengwen Zhou, Rongchun Liu.

**Software:** Jin Wen.

**Writing – original draft:** Hongxiu Yu.

**Writing – review & editing:** Ruixiang Chen.

## References

[1] Kaushansky K. Thrombopoietin: the primary regulator of platelet production. Blood. 1995;86:419–31. 7605981

[2] Franco AT Ranco AT Corken A. Platelets at the interface of thrombosis, inflammation, and cancer. Blood. 2015;126:582–8. 26109205 10.1182/blood-2014-08-531582 PMC4520875

[3] Lee EJ Lee AI. Thrombocytopenia. Prim Care 2016;43:543–57. 27866576 10.1016/j.pop.2016.07.008

[4] Crowther MA Cook DJ Meade MO. Thrombocytopenia in medical-surgical critically ill patients: prevalence, incidence, and risk factors. J Crit Care. 2005;20:348–53. 16310606 10.1016/j.jcrc.2005.09.008

[5] Khan AM Mydr AH Nevarez A. Clinical practice updates in the management of immune thrombocytopenia. P T 2017;42:756–63. 29234214 PMC5720488

[6] Provan D Arnold DM Bussel JB. Updated international consensus report on the investigation and management of primary immune thrombocytopenia. Blood Adv. 2019;3:3780–817. 31770441 10.1182/bloodadvances.2019000812 PMC6880896

[7] Neunert C Terrell DR Arnold DM. American Society of Hematology 2019 guidelines for immune thrombocytopenia. Blood Adv. 2019;3:3829–3866. Blood Adv. 2020;4:252. 31794604 10.1182/bloodadvances.2019000966 PMC6963252

[8] Wojciechowski P Wilson K Nazir J. Efficacy and safety of avatrombopag in patients with chronic immune thrombocytopenia: a systematic literature review and network meta-analysis. Adv Ther. 2021;38:3113–28. 33934279 10.1007/s12325-021-01752-4 PMC8189936

[9] Xu RH Shi YK Feng JF. Consensus on the clinical diagnosis, treatment, and prevention of chemotherapy-induced thrombocytopenia in China (2019 version). Chin J Front Med. 2020;12:51–8.

[10] Silva T Oliveira C Borges F. Caffeic acid derivatives, analogs and applications: a patent review (2009-2013). Expert Opin Ther Pat 2014;24:1257–70. 25284760 10.1517/13543776.2014.959492

[11] Liu XY Zha DJ. Advances in pharmacological activity and molecular mechanism of caffeic acid phenethyl ester. J Fujian Med Univ. 2021;55:570–4.

[12] Sova M Saso L. Natural sources, pharmacokinetics, biological activities and health benefits of hydroxycinnamic acids and their metabolites. Nutrients. 2020;12. 10.3390/nu12082190 PMC7468728 32717940

[13] El-seedi HR El-said AMA Khalifa SAM. Biosynthesis, natural sources, dietary intake, pharmacokinetic properties, and biological activities of hydroxycinnamic acids. J Agric Food Chem. 2012;60:10877–95. 22931195 10.1021/jf301807g

[14] Delrod D Rodriguez-mateos A Spencer JPE. Dietary (Poly)phenolics in human health: structures, bioavailability, and evidence of protective effects against chronic diseases. Antioxid Redox Signal. 2013;18:1818–92. 22794138 10.1089/ars.2012.4581 PMC3619154

[15] Verma RP Hansch C. An Approach towards the quantitative structure-activity relationships of caffeic acid and its derivatives. ChemBioChem. 2004;5:1188–95. 15368569 10.1002/cbic.200400094

[16] Genaro-mattos TC Mauricio AQ Rettori D. antioxidant activity of caffeic acid against iron-induced free radical generation-A Chemical Approach. PLoS One. 2015;10:e0142402. 26098639 10.1371/journal.pone.0129963 PMC4476807

[17] Tosovic J. Spectroscopic features of caffic acid:theoretical study. Kragujevac J Sci. 2017;2017:99–108.

[18] Qin H Yu H Yan Y. Caffeic acid production enhancement by engineering a phenylalanine over-producingEscherichia colistrain. Biotechnol Bioeng. 2013;110:3188–96. 23801069 10.1002/bit.24988

[19] Lin Y Yan Y. Biosynthesis of caffeic acid in Escherichia coli using its endogenous hydroxylase complex. Microb Cell Fact. 2012;11:42. 22475509 10.1186/1475-2859-11-42 PMC3379962

[20] Rodrigues JL Araújo RG Prather KLJ. Heterologous production of caffeic acid from tyrosine in Escherichia coli. Enzyme Microb Technol. 2015;71:36–44. 25765308 10.1016/j.enzmictec.2015.01.001

[21] Kilani-Jaziri S Mokdad-Bzeouich I Kriea M. Immunomodulatory and cellular anti-oxidant activities of caffeic, ferulic, and p-coumaric phenolic acids: a structure-activity relationship study. Drug Chem Toxicol. 2017;40:416–24. 27855523 10.1080/01480545.2016.1252919

[22] Nagaoka T Banskota AH Tezuka Y. Selective antiproliferative activity of caffeic acid phenethyl ester analogues on highly liver-metastatic murine colon 26-L5 carcinoma cell line. Bioorg Med Chem. 2002;10:3351–9. 12150882 10.1016/s0968-0896(02)00138-4

[23] Xie J Yang F Zhang M. Antiproliferative activity and SARs of caffeic acid esters with mono-substituted phenylethanols moiety. Bioorg Med Chem Lett. 2017;27:131–4. 27979593 10.1016/j.bmcl.2016.12.007

[24] Bispo VS Dantas LS Chaves AF. Reduction of the DNA damages, hepatoprotective effect and antioxidant potential of the coconut water, ascorbic and caffeic acids in oxidative stress mediated by ethanol. An Acad Bras Cienc. 2017;89:1095–109. 28513780 10.1590/0001-3765201720160581

[25] Yang SY Hong CO Lee GP. The hepatoprotection of caffeic acid and rosmarinic acid, major compounds of Perilla frutescens, against t-BHP-induced oxidative liver damage. Food Chem Toxicol. 2013;55:92–9. 23306788 10.1016/j.fct.2012.12.042

[26] Gu W Yang Y Zhang C. Caffeic acid attenuates the angiogenic function of hepatocellular carcinoma cells via reduction in JNK-1-mediated HIF-1αstabilization in hypoxia. RSC Adv. 2016;6:82774–82.

[27] Won C Lee CS Lee JK. CADPE suppresses cyclin D1 expression in hepatocellular carcinoma by blocking IL-6-induced STAT3 activation. Anticancer Res. 2010;30:481–8. 20332458

[28] Lee KW Kang NJ Kim JH. Caffeic acid phenethyl ester inhibits invasion and expression of matrix metalloproteinase in SK-Hep1 human hepatocellular carcinoma cells by targeting nuclear factor kappa B. Genes Nutr. 2008;2:319–22. 18850224 10.1007/s12263-007-0067-9 PMC2478489

[29] Espindola K Ferreira RG Narvaea L. Chemical and pharmacological aspects of caffeic acid and its activity in hepatocarcinoma. Front Oncol. 2019;9:541. 31293975 10.3389/fonc.2019.00541 PMC6598430

[30] Yang JL Zhu XL Li CW. Progress on the pharmacological effects of caffeic acid and its derivative phenethyl caffeate. Chin Pharm J 2013;48:577–82.

[31] Moher D Liberati A Tetzlaff J. Preferred reporting items for systematic reviews and meta-analyses: the PRISMA statement. PLoS Med. 2009;6:e1000097. 19621072 10.1371/journal.pmed.1000097 PMC2707599

[32] Goodyear MD Krleza-Jeric K Lemmens T. The Declaration of Helsinki. BMJ. 2007;335:624–5. 17901471 10.1136/bmj.39339.610000.BE PMC1995496

[33] Wang Y. Introduction to the cochrane risk of biase assessment tool. Chin Gen Pract. 2019;22:1322.

[34] Higgins JP Altman DG Gqtzsche PC. The cochrane collaboration’s tool for assessing risk of bias in randomised trials. BMJ. 2011;343:d5928. 22008217 10.1136/bmj.d5928 PMC3196245

[35] Bei YX. Effect of caffeic acid tablets in the treatment of idiopathic thrombocytopenic purpura. Chin J Tralima Disability Medici. 2015;23:105–6.

[36] Chen M. Combination of prednisone caffeic acid tablets for idiopathic thrombocytopenic purpura. Guide China Med. 2012;10:520–1.

[37] Dong HZ Peng ZQ Wang MJ. Clinical observation of neutrophils and thrombocytopenia caused by chemotherapy causedby caffeic acid tablets. Jiangxi Med J. 2020;55:1825–6.

[38] Du ZM Jia HP Yang LL. Clinical study of caffeic acid against bone marrow suppression after chemotherapy for B-cell non-Hodgkin’s lymphoma. Chin J Pract Med. 2018;45:85–8791.

[39] Du ZM Chen HL Yang LL. Clinical observation of caffeic acid in preventing hemocytopenia in patients with lung cancer after radiotherapy. Clin Med china 2018;34:517–9.

[40] He D Li B Zhu XL. Clinical effect and safety of caffeic acid tablets in the treatment of immune thrombocytopenia. J Clin Hematol 2020;33:344–7.

[41] Huang ZH. Effect of caffeic acid on parameters and aggregation of the platelet in patients with uremic bleeding. Int Med China 2013;8:459–60.

[42] Jia XH Sun SB Yin WY. Observation of the curative effect of caffeic acid tablets in the treatment of systemic lupus erythematosus with leukocytes and thrombocytopenia: the seventh annual meeting of Hematology Branch of Hebei Medical Association and the fourth annual meeting of Hematology Branch of Hebei Medical Association[C]. Tangshan, 2013.

[43] Kang JD. Clinical application of caffeic acid tablet for treating idiopathic thrombocytopenic purpura. Med Info. 2013;1:129.

[44] Lin W Zhu HF Li LJ. Effects of different dose of caffeic acid tablets combined with rhIL-11 on prevention of chemotherapy-induced thrombocytopenia in leukemia patients. J Big Doctors. 2022;7:4–7.

[45] Lin WY Chen BL Mo DH. Efficacy of caffeic acid tablets in the treatment of idiopathic thrombocytopenic purpura. J Pract Med. 2009;25:3491–2.

[46] Liu D. Clinical effect of caffeic acid tablets on chronic refractory ITP. Med Info. 2015;11:375.

[47] Liu F Xiao DH Mo DH. Cinlincal observation of caffeic acid tablet in treatment of alpastic anemia. Clin Ration Drug Use. 2011;4:13–5.

[48] Liu K Ruan LH. Amoxiciilin, the coffee acid treated first joint prednisone treatment of idiopathic thrombocytopenic purpura clinical research. Chin Med Inno. 2012;9:24–5.

[49] Liu XR. Effect of caffeic acid tablets in esophageal cancer. Hebei Med J. 2016;38:678–80.

[50] Li XS Du J. Analysis of caffeic acid combined with HP for chronic non-severe ITP. Chin Med Guide. 2013;11:11–2.

[51] Li XH Zhang QQ Li J. Caffeic acid tablets combined with traditional Chinese medicine treated 62 cases of refractory immune thrombocytopenia. Chin J Gerontol. 2013;33:2938–9.

[52] Luo YH Liu DH Lian HY. The curative effect of leukocyte and thrombocytopenia caused by hyperactivityin cirrhosis. Chin Foreign Med Res. 2013;11:39–40.

[53] Ma J Shao XR Shen ZX. A randomized, double-blind multicentre clinical trial comparing the efficacy and safety of Caffeic acid for the treatment of immune thrombocytopenia in China. Chin J Pract Int Med. 2017;37:817–21.

[54] Meng DQ Wang GR Li J. Efficacy of caffeic acid tablets combined with prednisone and hydroxychloroquine in the treatment of systemic lupus erythematosus. Chin J Clin Res. 2018;31:1401–4.

[55] Meng DQ Li RS Li J. Clinical efficacy of caffeic acid tablet combined with traditional immunotherapy in the treatment of blood system damage in Sjogren’s syndrome. China Med Pharm. 2021;11:192–5.

[56] Miao D Yang L. Clinical observation of cyclosporine combined with caffeic acid in the treatment of refractory idiopathic thrombocytopenic purpura. J Medical Forum 2013;34:116–7.

[57] Pan B Yu Y Hu J. Clinical study of Kafeisuan tablet for prevention of chemotherapy induced thrombocytopenia. Mod Oncol Med 2013;21:2817–9.

[58] Pang Y Feng Y Ye X. Clinical study of caffeic acid in the treatment of leukopenia and thrombocytopenia after chemotherapy in non-Hodgkin’s lymphoma. J Clin Hematol (China) 2012;25:461–2.

[59] Peng AH. Clinical study of caffeic acid tablets in the early intervention of adjuvant chemotherapy after breast cancer surgery[D]. Oncology, Guangxi University of Traditional Chinese Medicine, 2015.

[60] Tang TB Wang JP Wu D. Clinical observation of caffeic acid in the treatment of leukopenia and thrombocytopenia caused by antituberculosis drugs. J Ningxia Med Univ 2017;39:78–80.

[61] Wang HC Xu NL Qin XY. To investigate the effect of caffeic acid tablets on leukopenia induced by postoperative chemotherapy for breast cancer. Chin J Mod Drug Appl 2016;10:162–3.

[62] Wang J Chen BA Ding JH. Efficacy of caffeic acid tablets combined with corticoids on primary immune thrombocytopenia. Jiangsu Med J 2013;39:2143–4.

[63] Xin XH. Efficacy of caffeic acid tablets in preventing chemotherapy-induced thrombocytopenia. China Med Eng. 2015;23:184.

[64] Xu D Ji CF Chen C. The Effect of Dexamethasone Combinated with Caffeic Acid Tablet on PAIgG and GPIIb/IIIa Levels in Patients with Primary Immune Thrombocytopenia. J Clin Transfus Lab Med 2020;20:3–5.

[65] Yang X Jiao R Huang W. Effect of caffeic acid tablets in pediatric immune thrombocytopenia. Chin Foreign Med Res. 2019;17:135–6.

[66] Zhang XM. Clinical study of caffeic acid for the treatment of bone marrow suppression after chemotherapy for hematological tumors in children. J Health Lit. 2022;23:27–8.

[67] Zhang XJ Wang CX Quan SZ. Clinical study on 33 cases of immunoinduced thrombocytopenia. Chin J Clin Rational Drug Use. 2013;6:3–4.

[68] Zhao BF. Analysis of the effect of caffeic acid against leukocytes and thrombocytopenia after chemotherapy in lung cancer. New Med J. 2017;27:291–2.

[69] Zhou CX. Effect of combined therapy on chronic aplastic anemia. Advice Health 2022;16:29–3137.

[70] Yuan XL. Chinese expert consensus on management of thrombocytopenia in cancer patients with liver injury (2022 edition). J Clin Hepatol. 2023;39:1287–94.

[71] Haemostasis and Thrombosis Group of the Haematology Branch of the Chinese Medical Association. Chinese expert consensus on the diagnosis and treatment of primary immune thrombocytopenia in adults (2016 edition). Chin J Haematol. 2016;37:89–93.

[72] Wu Y Aravind S Ranganathan G. Anemia and thrombocytopenia in patients undergoing chemotherapy for solid tumors: a descriptive study of a large outpatient oncology practice database, 2000-2007. Clin Ther. 2009;31 Pt 2(Pt 2):2416–32. 20110050 10.1016/j.clinthera.2009.11.020

[73] Paciello F Dipion A Rolesi R. Anti-oxidant and anti-inflammatory effects of caffeic acid: in vivo evidences in a model of noise-induced hearing loss. Food Chem Toxicol. 2020;143:111555. 32640333 10.1016/j.fct.2020.111555

[74] Qi J. Clinical study of caffeic acid tablets in the treatment of leukopenia induced by chemotherapy for cervical cancer. J Hunan Normal Univ 2019;16:92–6.

[75] Wang X Li W Ma X. Simultaneous determination of caffeic acid and its major pharmacologically active metabolites in rat plasma by LC-MS/MS and its application in pharmacokinetic study. Biomed Chromatogr. 2015;29:552–9. 25164780 10.1002/bmc.3313

[76] Qin P Wei Y Hou M. A multicenter clinical trial of caffeic acid tablet in treatment of 103 primary immune thrombocytopenia patients. Chin J Hematol 2015;36:103–6. 10.3760/cma.j.issn.0253-2727.2015.02.004 PMC7342146 25778883

[77] Wang J Liu ZJ Kang WY. Effect of caffeic acid tablets combined with recombinant human granulocyte colony-stimulating factor on severe myelosuppression after chemotherapy for cervical cancer. Chin J SurgOncol 2019;11:4.

[78] Liang Y Feng G Wu L. Caffeic acid phenethyl ester suppressed growth and metastasis of nasopharyngeal carcinoma cells by inactivating the NF-κB pathway. Drug Des Devel Ther 2019;13:1335–45. 10.2147/DDDT.S199182 PMC6499142 31118570

